# Spatially Modulated Irradiation Alters Extracellular Vesicle MicroRNA Cargo in Rat Glioma and Astrocyte Models

**DOI:** 10.1007/s12035-026-05988-5

**Published:** 2026-06-11

**Authors:** Sarah Potiron, Julie Espenon, Cristele Gilbert, Ahmed Glayl, Alfredo Fernandez-Rodriguez, Angela Corvino, Marjorie Juchaux, Yolanda Prezado

**Affiliations:** 1 Institut Curie, Université PSL, CNRS, UMR3347, Inserm U1021, Signalisation Radiobiologie Et Cancer, Orsay, France; 2 Université Paris-Saclay, CNRS, UMR3347, Inserm U1021, Signalisation Radiobiologie Et Cancer, Orsay, France; 3 New Approaches in Radiotherapy Lab, Center for Research in Molecular Medicine and Chronic Diseases (CIMUS), Instituto de Investigación Sanitaria de Santiago de Compostela (IDIS), University of Santiago de Compostela, A Coruña, Spain; 4 Oportunius Program, Galician Agency of Innovation (GAIN), Xunta de Galicia, A Coruña, Spain

**Keywords:** Minibeam radiation therapy, Conventional radiotherapy, Extracellular vesicles

## Abstract

Spatially fractionated radiation therapy, particularly minibeam radiation therapy (MBRT), improves normal tissue sparing while maintaining tumor control compared with conventional radiotherapy (CRT). However, the mechanisms underlying these advantages remain unclear. EVs carrying miRNAs are increasingly recognized as mediators of radiation-induced intercellular communication and may contribute to non-targeted and cohort effects. We investigated the impact of MBRT versus CRT on EV-miRNA profiles in glioma and normal astrocyte cell lines. Rat glioma (RG2, F98) and astrocyte (CTX-TNA2) cell lines were irradiated with X-ray CRT or MBRT. Small EVs were isolated 24 h post-irradiation, characterized by transmission electron microscopy and nanoflow cytometry, and analyzed by small RNA sequencing. EV morphology, size, and expression of canonical markers (CD9, CD63, CD81) were preserved across conditions, indicating that irradiation primarily altered EV cargo. Glioma cells showed limited and cell-dependent changes. In RG2 cells, CRT increased miR-6319 and miR-374-5p, whereas MBRT modulated miR-151-5p in a dose-dependent manner and reduced miR-466c-5p at 5 Gy. No significant changes were observed in F98 cells. In contrast, astrocytes exhibited marked remodeling of EV-miRNA cargo after irradiation, including enrichment of miRNAs associated with stress adaptation, hypoxia-related signalling, and immune regulation, together with consistent depletion of miR-122b and miR-486 across irradiation conditions. MBRT and CRT were associated with differential remodeling of EV miRNA cargo, with a markedly stronger response in astrocytes than in glioma cells. These findings suggest altered EV signaling potential following irradiation and provide an initial molecular framework for investigating how spatial dose modulation may influence extracellular communication. Further functional validation in human-relevant systems is required to determine mechanistic and translational significance.

**Supplementary Information:**

The online version contains supplementary material available at 10.1007/s12035-026-05988-5.

## Introduction

Radiotherapy (RT) is a pivotal component in cancer treatment. The clinical efficacy of RT has traditionally been attributed to the local effects of ionizing radiation, which results in cell death by directly and indirectly inducing DNA damage, but substantial work has highlighted the importance of non-targeted effects [1]. Numerous studies have shown that signals released by irradiated cells can influence neighboring non-irradiated cells, inducing radiation-like biological responses [1–3]. These phenomena are collectively referred to as non-targeted effects (NTEs), including bystander, abscopal, and cohort effects, the latter being the least investigated [1–3].

Various substances, including cytokines, reactive oxygen species, and extracellular vesicles (EVs), are implicated in these radiation-induced intercellular communication pathways, affecting biological processes, such as oxidative stress, immune modulation, DNA damage and repair, and gene expression [4–6]. Among these, EVs have become important mediators of non-targeted responses, transporting bioactive substances from irradiated cells to distant bystander cells, such as proteins, lipids, and RNA species [7, 8], inducing gene expression modulation. Extracellular vesicles (EVs) comprise a heterogeneous population that includes exosomes, microvesicles, and non-vesicular nanoparticles such as exomeres [9]. Within this diversity, small extracellular vesicles (sEVs), typically ranging from approximately 30 to 200 nm in size [9–11], originate from the endosomal pathway and are released into the extracellular matrix following the fusion of multivesicular bodies with the plasma membrane [12, 13]. These vesicles have been shown to mediate radiation-induced alterations in the tumor microenvironment (TME), modulating immune responses and tumor progression [14]. sEVs contribute to processes such as angiogenesis, formation of the pre-metastatic niche, and the development of radioresistance in various cancer types [15–18].

Among the molecular cargo of sEVs, microRNAs (miRNAs), small non-coding RNAs that regulate gene expression post-transcriptionally [19], have drawn particular attention. Radiation has been shown to modulate miRNA expression [20, 21], and miRNA-containing sEVs have been implicated in transmitting radiation-induced biological effects (RIBEs) [22–24]. For instance, low-dose radiation has been shown to induce EV release in glioblastoma cell lines, which can confer resistance to RT through enhanced DNA repair pathways [25]. In several other cancers, sEVs-miRNAs have been associated with radioresistance [26, 27], suggesting their potential utility as biomarkers for prognosis, diagnosis, and therapy monitoring in glioblastoma [28, 29].

The cohort effect, a form of NTEs, refers to the interaction between irradiated cells within a spatially heterogeneous irradiated volume, where cells receiving high doses of radiation influence those receiving low doses, and vice versa [30]. This effect is spatially limited, typically confined to a few millimeters within the irradiated tissue and may play an important role in modulating both tumor response and normal tissue protection in spatially fractionated radiation therapy (SFRT) [31–33].

SFRT is an unconventional form of RT characterized by spatial dose modulation, where alternating regions of high-dose “peaks” and low-dose “valleys” are delivered to the target volume [31]. Despite this heterogeneous dose distribution, SFRT has demonstrated both remarkable tumor control and significant normal tissue sparing [31, 34–36]. However, the underlying molecular mechanisms driving these responses remain poorly understood.

This pilot study aims to investigate the biological processes associated with SFRT, with particular interest in its unexplored impact on EV biology. Specifically, we concentrate on minibeam radiation therapy (MBRT), a form of SFRT that delivers narrow, spatially fractionated beams [37, 38]. MBRT has been shown to enhance tissue sparing [39–42] and reduce neurotoxicity [40, 41, 43, 44], while providing a strong antitumor effect [45–50], making it a promising strategy for treating challenging tumors such as glioblastomas [45–49]. Recent evidence has further suggested that MBRT-induced responses are immune-mediated, promoting T cell infiltration and antitumor immunity [49, 51].

In this study, we report for the first time the characterization of sEVs secretion and their miRNA cargo in both glioblastoma and normal astrocyte cell lines following treatment with MBRT or conventional RT (CRT), using a deep sequencing approach. This work seeks to unravel novel insights into the intercellular communication mechanisms triggered by spatially modulated irradiation.

## Material and Methods

### Cell Culture

Three rat cell lines were employed for this study: two glioma cell lines, RG2 [D74] (ATCC, CRL-2433, RRID: CVCL_3581) and F98 (ATCC, CRL-2397, RRID: CVCL_3510), and one rat astrocyte cell line, CTX-TNA2 (ECACC, 98102213, RRID: CVCL_3670).

RG2 and F98 cells were cultured in Dulbecco’s Modified Eagle Medium (DMEM; Gibco, Thermo Fisher Scientific, Cat. No. 41965-039) supplemented with 1 mM sodium pyruvate (Gibco, Cat. No. 11360-039), 10% fetal bovine serum (FBS; Gibco, Cat. No. 10270-106), 1 × Fungizone™ Antimycotic (amphotericin B, 1.25 µg/mL final; Gibco, Cat. No. 15290-018), and 1% penicillin–streptomycin (100 U/mL penicillin and 100 µg/mL streptomycin; Gibco, Cat. No. 15140-122). Additionally, RG2 medium was supplemented with 1 mM HEPES (Gibco, Cat. No. 15630-056). CTX-TNA2 cells were cultured in DMEM supplemented with 2 mM L-glutamine (Gibco, Cat. No. 25030-024), 10% FBS, 1 × Fungizone™ Antimycotic, and 1% penicillin–streptomycin. Cells were maintained as monolayer cultures in tissue culture flasks, kept at 37 °C in an incubator with humidified air supplemented with 5% CO_2_. Cells were routinely screened for *Mycoplasma* using the Mycoplasma Detection Kit (Sigma-Aldrich, Cat. No. MP-0035-1KT).

For all experiments, cells were routinely cultured and passaged twice a week. Cells were plated 48 h or 72 h before X-ray irradiation to achieve 70–80% confluency on the day of the irradiation.

Medium was replaced 1 h before irradiation in all experimental and sham-irradiated control groups to standardize baseline culture conditions and define a consistent 24-h conditioned-medium collection window for EV isolation.

### Irradiations

Irradiations were carried out with X-rays at a Small Animal Radiation Research Platform (SARRP, Xstrahl Ltd, UK). Irradiations were performed in three independent biological replicates (independent cell culture preparations/passages) for each condition. Non-irradiated controls underwent sham irradiation, including identical transport, handling, and placement in the irradiation setup without radiation delivery. X-rays were generated by means of mechanical brass collimators. The X-ray minibeams were 0.7 mm wide, with a center-to-center (ctc) distance of 1.4 mm.

Mean doses of 5, 10, and 20 Grays (Gy) were delivered to the tumor cell lines (RG2, F98), which required higher doses due to their high radioresistance. The normal astrocyte cell line (CTX-TNA2) was irradiated with 2, 5, and 10 Gy mean doses. Gafchromic films were used for dose measurement and quality control of irradiations, and the correspondence between the mean doses with the peak and valley doses in X-ray MBRT are described in Table 1 for each condition.

**Table 1:** Correspondence between mean, peak, and valley doses in X-ray MBRT

	X-ray MBRT	
Mean dose	Peak dose	Valley dose
2.0 ± 0.1 Gy	4.2 ± 0.3 Gy	0.63 ± 0.05 Gy
5.0 ± 0.4 Gy	9.5 ± 0.5 Gy	1.30 ± 0.06 Gy
10.0 ± 0.8 Gy	22 ± 1 Gy	2.7 ± 0.3 Gy
20.0 ± 1.6 Gy	44 ± 1 Gy	4.5 ± 0.5 Gy

### Small Extracellular Vesicles (sEVs) Isolation

Conditioned cell culture medium was collected 24 h post-irradiation (hpi) and centrifuged at 350×*g* for 15 min at 4 °C, followed by 2000×*g* for 20 min at 4 °C to remove cellular debris and dead cells. For EV-specific experiments, culture medium was supplemented with exosome-depleted FBS (System Biosciences, Cat. No. EXO-FBS-50A-1) to avoid contamination by bovine-derived vesicles. The supernatant was incubated with one-half volume of Total Exosome Isolation Reagent (from cell culture media; Invitrogen, Thermo Fisher Scientific, Cat. No. 4478359), thoroughly mixed, and incubated overnight at 4 °C. A final centrifugation step at 10,000×*g* for 1 h at 4 °C was then performed to pellet sEVs. The supernatant was discarded, and the exosomal pellet was resuspended in 100 μL of sterile 0.22-μm-filtered PBS (ClearLine, Cat. No. 146560). sEVs were stored at −80 °C until downstream analysis. sEV preparations were characterized according to MISEV guidelines using orthogonal approaches, including transmission electron microscopy (TEM) and nanoflow cytometry (nFCM**)** [10, 11].

### Negative Staining and Transmission Electron Microscopy (TEM) Imaging

For each sample, 4 µL of the sEV suspension was deposited onto carbon-coated copper grids (300 mesh; Ted Pella Inc., Cat. No. 0753-F) and allowed to adsorb for 5 min. During this time, 30 µL of 2% uranyl acetate solution (Electron Microscopy Sciences, Cat. No. 22400) was prepared and protected from light to serve as a negative contrast stain. Excess liquid was gently blotted from the edges of the grid using filter paper, after which the carbon-coated side was placed on a drop of uranyl acetate for 20 s in the dark to enhance contrast. Following staining, excess uranyl acetate was removed, and the grids were air-dried prior to imaging. sEVs were imaged using a Hitachi HT7700 transmission electron microscope (120 kV; tungsten filament) operated at 80 keV and 10 µA under vacuum conditions. High-resolution micrographs were acquired at ×20,000 and ×30,000 magnification at multiple locations across the grid. A minimum of 100 sEVs were measured across multiple fields of view, to obtain a representative size distribution of the sample.

### Nanoflow Cytometry (nFCM)

Nanoflow cytometry was performed using a NanoAnalyzer instrument (NanoFCM Inc., Xiamen, China) equipped with high-sensitivity detectors for small particle analysis. EV size and concentration were determined prior to antibody staining using calibration nanosphere standards (40–200 nm; NanoFCM Inc., Xiamen, China) to define the size detection range. EVs were then stained with fluorescently conjugated antibodies against the canonical tetraspanins CD9, CD63, and CD81: FITC anti-rat CD9 (clone 2A1/CD9, BioLegend, Cat. No. 206506, RRID: AB_2894442), Alexa Fluor 647 anti-rat CD63 (clone AD1, Bio-Rad, Cat. No. MCA475A-A647, RRID: AB_10613490), and PE anti-mouse/rat CD81 (clone EAT-2, BioLegend, Cat. No. 104906, RRID: AB_2076266). For each staining reaction, 9 μL of concentrated EV suspension was incubated with 1 μL of antibody solution for 30 min at 4 °C, protected from light. Due to the two-channel configuration of the nFCM instrument, samples were analyzed using two dual-color staining panels. Following incubation, unbound antibodies were removed using Exo-spin™ columns (Cell Guidance Systems, Cat. No. EXS-5) according to the manufacturer’s instructions. Purified EVs were then resuspended in 100 μL of PBS, and each sample was acquired on the nFCM system for 90 s.

### RNA isolation

Total RNA, including small RNA species such as microRNAs, was extracted from extracellular vesicles using the miRNeasy Mini Kit (Qiagen, Cat. No. 217004), following manufacturer’s instructions with slight modifications. Briefly, EVs-containing samples were lysed in QIAzol Lysis Reagent (Qiagen, Cat. No. 79306) and homogenized. After incubation, chloroform was added, and phase separation was achieved by centrifugation at 12,000×*g* for 15 min at 4 °C. The aqueous phase, containing RNA, was carefully transferred to a new tube and mixed with 1.5 volumes of ethanol to facilitate RNA binding to the spin column membrane. The lysate-ethanol mixture was loaded onto the miRNeasy column and centrifuged at 12,000×*g* for 30 s. The membrane was then sequentially washed and RNA eluted in RNase-free water by centrifugation and quantified using a NanoDrop spectrometer (Thermo Fisher Scientific). The extracted RNA was immediately stored at −80 °C.

### Library Preparation, Next-Generation Small RNA Sequencing, and Bio-Informatics Analysis

After RNA extraction, quality and RNA concentrations were obtained using RNA 6000 Pico Kit (Agilent Technologies, Cat. No. 5067-1513) for 2100 Bioanalyzer Systems (Agilent Technologies) as per the manufacturer’s instructions. To construct the libraries, 1–5 ng of the small RNA fraction was processed using QIAseq miRNA Library Kit (Qiagen, Cat. No. 331502) according to manufacturer’s instructions. Briefly, 3’ and 5’ adapters were ligated to each end of RNA molecules and then reverse transcribed and amplified to generate a cDNA library. Each library is then controlled on a High Sensitivity DNA chip (Agilent Technologies). Libraries were sequenced on an Illumina NextSeq500 instrument using 75 base-lengths read in a single read mode. After sequencing, a primary analysis based on AOZAN software (ENS, Paris) was applied to demultiplex and control the quality of the raw data (based on FastQC modules/version 0.11.5).

The raw sequencing data have been processed with the nf-core small RNA-seq pipeline (version 2.3.1.) available at https://nf-co.re/smrnaseq/2.3.1 [52]. Nf-core/smrnaseq is a bioinformatics best-practice analysis pipeline for small RNA sequencing built using Nextflow for running quality controls and adapter trimming on the raw sequencing reads to thus align them on the rat miRbase (Rnor 6.0) database to quantify both mature and hairpin miRNAs.

Raw counts were normalized with the TMM method from edgeR [53] which computes scaling factors to correct compositional biases between samples. Lowly expressed miRNAs, defined as not having more than 3 cpm (count per million) in at least three samples, were then filtered out. Differential analysis was then carried out using the limma/voom framework [54, 55], incorporating random effects to account for biological replicate variability. The resulting *p*-values were corrected by Benjamini–Hochberg procedure, and miRNAs with adjusted *p*-values below 0.05 were declared differentially expressed. All analyses were conducted in R software version 3.4.1. [56] in the bioinformatics platform at Institut Curie (CurieCoreTech, Paris, France).

## Results

This section presents the results obtained regarding the isolation and characterization of sEVs from the cell culture supernatant of the different cell lines considered in the study and the sequencing of the miRNA purified from the sEVs.

### Characterization of Isolated sEVs Before Transcriptomic Analysis

To evaluate the specific miRNA signatures of each cell line after irradiation, an initial characterization of the sEVs isolated from them was required in order to validate the nature and quality. This characterization is recommended under MISEV guidelines to support rigorous EV reporting [10, 11].

**Fig. 1:**
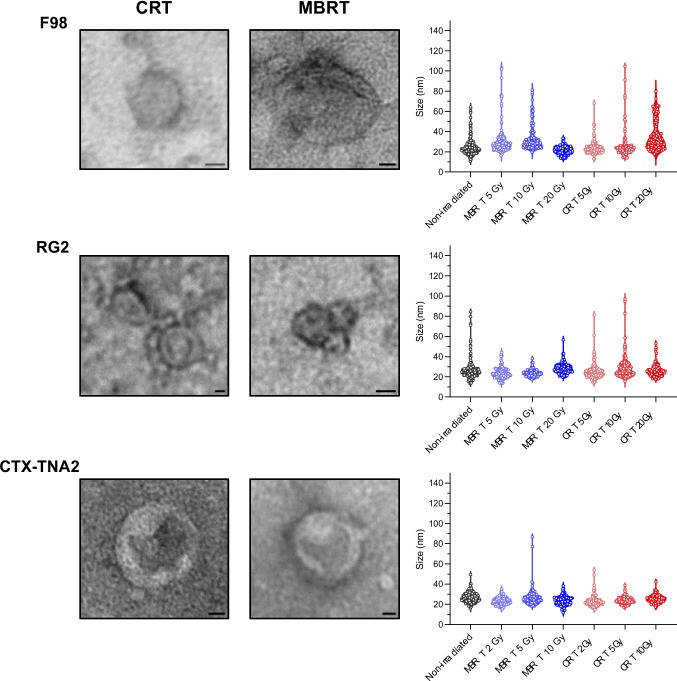
Characterization of isolated small extracellular vesicles (sEVs). Left: Representative TEM images showing the morphology of isolated sEVs collected 24 h post-irradiation following a single 10 Gy exposure by CRT or MBRT. EVs were visualized at different locations on the grid, with over 100 EVs counted per sample to ensure a representative size distribution. Scale bars = 20 nm. Right: Representative nanoflow cytometry analysis of particle size distribution and concentration. TEM characterization was performed as descriptive morphological validation of EV preparations in accordance with MISEV recommendations and was not intended for formal comparative morphometric statistical analysis between irradiation conditions. Nanoflow measurements were obtained from *n* = 3 independent biological replicates per condition

At 24 hpi, sEVs were isolated from cell culture supernatants using a polymer-based precipitation method, followed by downstream characterization by TEM and nanoflow cytometry. First, the morphology and size representation were assessed by transmission electron microscopy imaging (Fig. 1). Representative TEM imaging showed round, cup-shaped vesicular structures with diameters spanning ~30–140 nm, consistent with sEV morphology. Occasional smaller nanoparticles (< 30–40 nm), compatible with exomere-sized particles, were also observed. In contrast, nFCM characterization was restricted to its validated detection range of 40–200 nm; therefore, particles below this threshold were not represented in nFCM size distributions. This initial characterization provided information about the structural integrity and general morphology of the sEVs isolated before proceeding with further characterization.

**Fig. 2:**
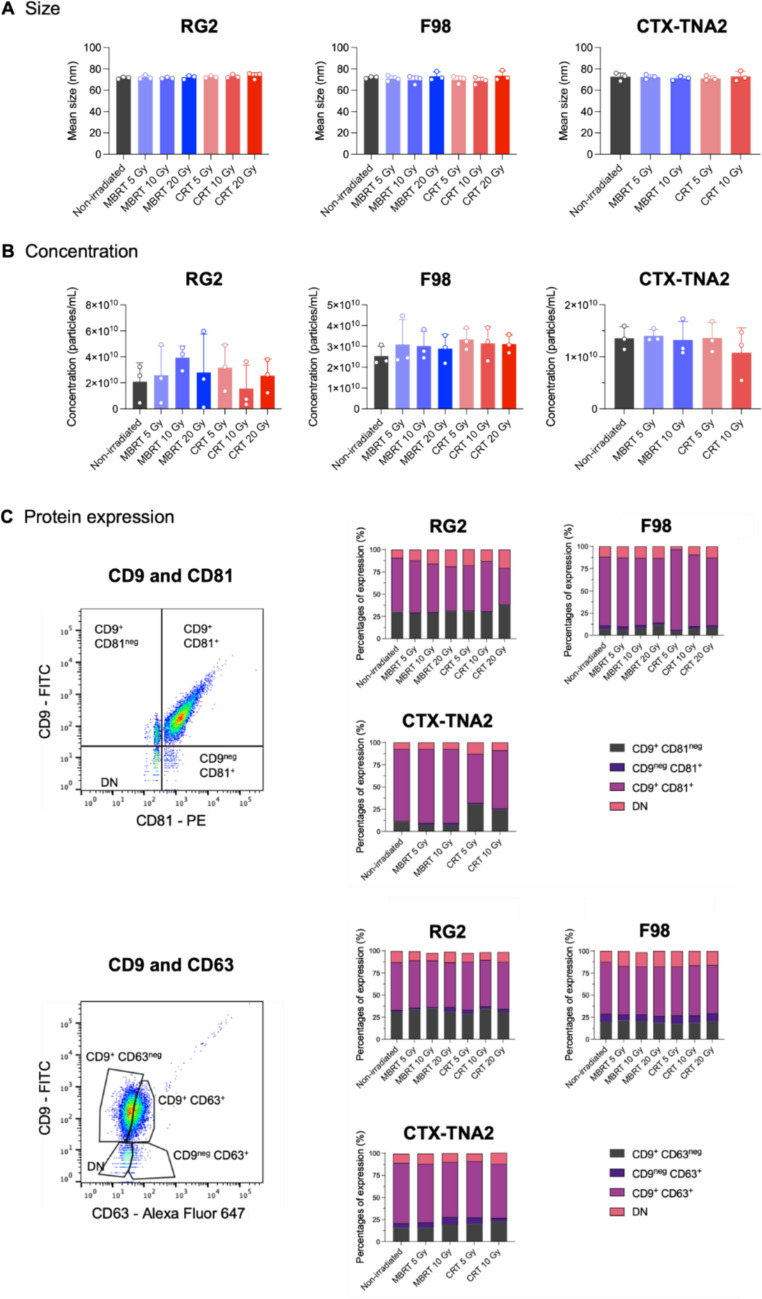
Phenotypic characterization and quantification of small extracellular vesicles (sEVs) by nanoflow cytometry (nFCM). **A** Size distribution of sEVs. **B** Particle concentration (particles/mL). **C** Surface protein phenotype based on tetraspanin expression (CD9, CD63, and CD81) assessed by dual-color immunolabeling using nFCM. The instrument was calibrated with reference beads ranging from 40 to 200 nm; therefore, particles outside this detection range were not measured. Protein expression was evaluated using two dual-color panels (CD9/CD81 and CD9/CD63) and is presented as the percentage of double-positive events. A representative staining profile is shown on the left of the graphs. Data were obtained from *n* = 3 independent biological replicates per condition

Then, to continue the characterization, sEVs were phenotypically evaluated by nFCM using fluorescent antibodies to the canonical tetraspanins CD9, CD63, and CD81. Across cell lines and conditions (irradiated and control), nFCM reported mean diameters of approximately 70 nm (Fig. 2A). Particle yields varied between biological replicates, from ~1 × 10^10^ to 6 × 10^10^ particles/mL (Fig. 2B), providing sufficient material for downstream analyses. Vesicular identity was confirmed by dual-color co-labeling in two panels (CD9 + CD81 and CD9 + CD63), with more than 50% of detected events double-positive in all preparations and only a minor tetraspanin-negative fraction (Fig. 2C). Collectively, these data verify that the isolates are enriched in *bona fide* sEVs with the expected size range and tetraspanin phenotype, supporting subsequent miRNA cargo profiling described below.

### Transcriptomic Analysis of the Small Non-coding miRNA Contained in sEVs After Irradiation

**Fig. 3:**
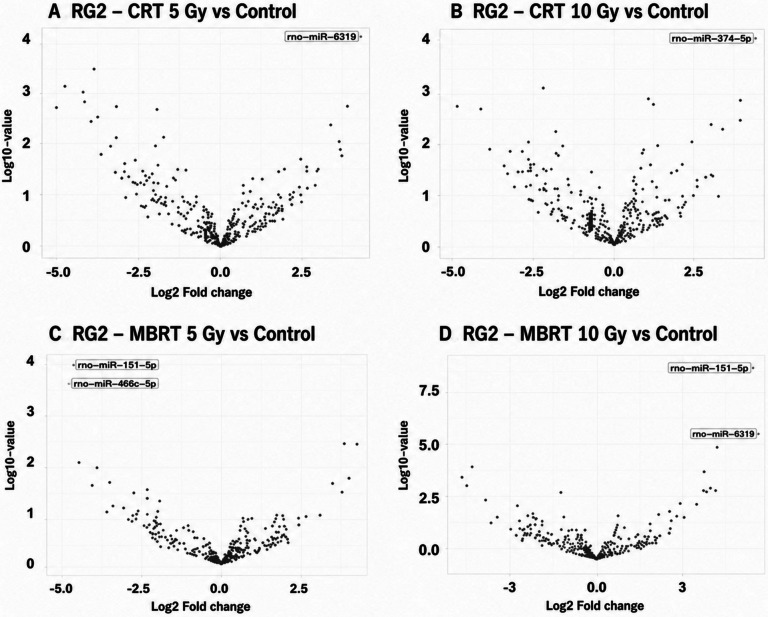
Differential EV-miRNA expression in glioma-derived extracellular vesicles following CRT and MBRT. Volcano plots showing differential expression of miRNAs isolated from small extracellular vesicles (sEVs) derived from the RG2 glioma cell line following irradiation. Comparisons are shown between non-irradiated controls and **A** CRT 5 Gy, **B** CRT 10 Gy, **C** MBRT 5 Gy, and **D** MBRT 10 Gy. Each dot represents a single miRNA. The *x*-axis indicates the log2 fold change in expression between groups, while the *y*-axis represents the − log10 adjusted *p* value. Red points denote significantly dysregulated miRNAs (upregulated on the right; downregulated on the left), defined by thresholds of |log2FC|≥ 1 and adjusted *p* < 0.05. Selected significantly altered miRNAs are annotated by name

Small RNA sequencing of sEVs released by glioma (RG2, F98) and non-cancerous astrocyte (CTX-TNA2) cell lines revealed limited changes overall, with no miRNAs meeting the significance threshold in F98 across conditions. In RG2-derived sEVs, modality- and dose-dependent differences emerged (Fig. 3 and Table S1 in supplemental materials). At 5 Gy, CRT increased miR-6319 relative to non-irradiated controls, while direct comparison between CRT and isodose MBRT identified differential modulation of miR-151-5p. At 10 Gy CRT, *miR-374-5p* was significantly enriched compared with controls. Conversely, MBRT at 5 Gy showed depletion of *miR-151-5p* and *miR-466c-5p* versus non-irradiated cells. Notably, *miR-6319* is sparsely annotated in the literature, limiting biological interpretation.

**Fig. 4:**
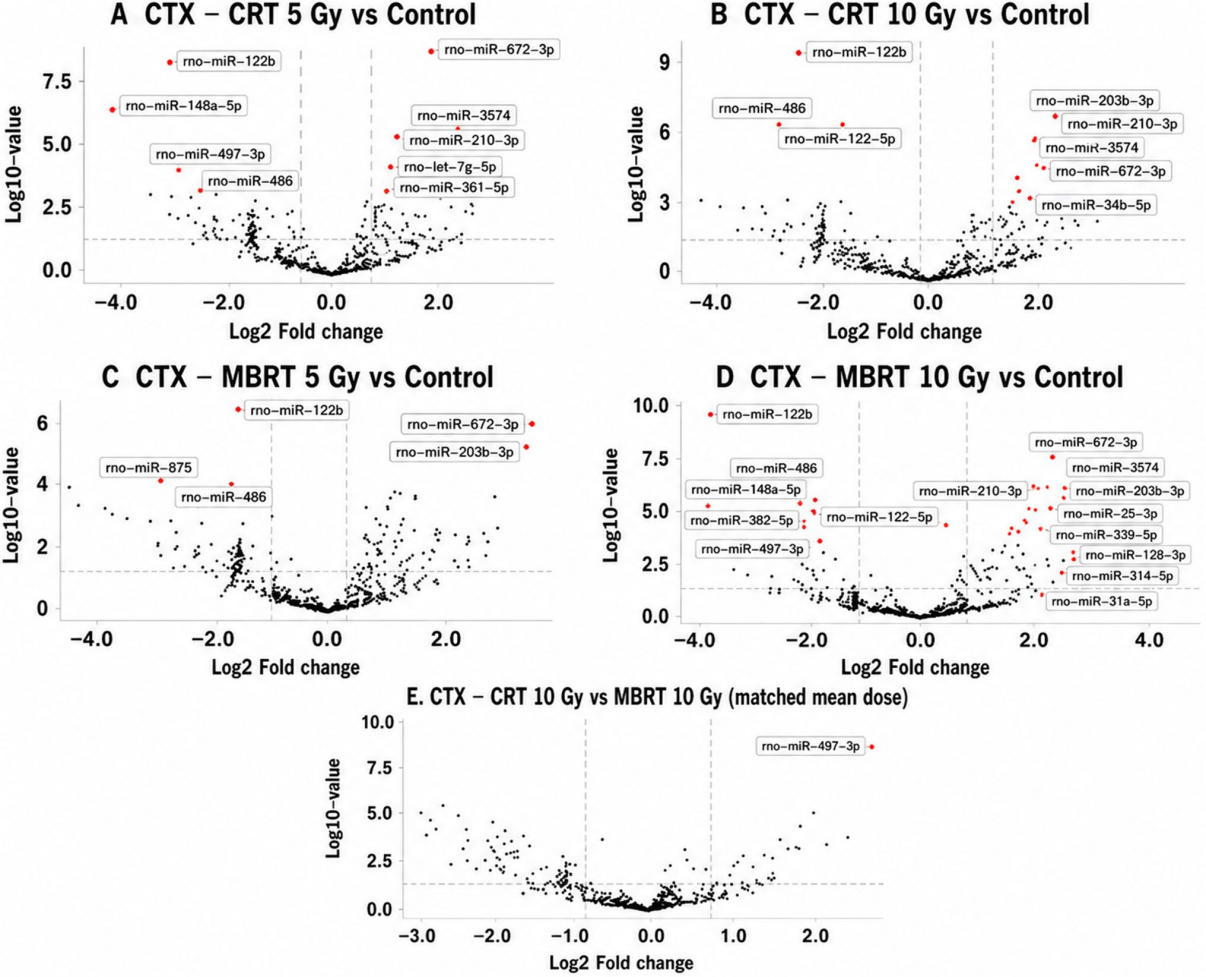
Differential EV-miRNA expression in astrocyte-derived extracellular vesicles following CRT and MBRT. Volcano plots showing differential expression of miRNAs isolated from small extracellular vesicles (sEVs) derived from the CTX-TNA2 astrocyte cell line 24 h after irradiation. Comparisons are shown between non-irradiated controls and **A** CRT 5 Gy, **B** CRT 10 Gy, **C** MBRT 5 Gy, and **D** MBRT 10 Gy, as well as the direct comparison between CRT and MBRT 10 Gy at matched mean dose (**E**). Each dot represents a single miRNA. The *x*-axis indicates the log2 fold change in expression between groups, while the *y*-axis represents the − log10 adjusted *p* value. Highlighted points denote significantly dysregulated miRNAs, defined by thresholds of |log2FC|≥ 1 and adjusted *p* < 0.05, with selected significantly altered miRNAs annotated by name

In CTX-TNA2-derived sEVs, irradiation induced marked remodeling of EV-miRNA cargo, with partially overlapping signatures across modalities and dose levels (Fig. 4 and Table S1). Several stress- and adaptive response–associated miRNAs, including miR-210-3p, miR-203b-3p, and miR-672-3p, were enriched in multiple irradiation conditions, whereas miR-122b and miR-486 showed consistent depletion across all conditions examined. Additional miRNAs, such as miR-148a-5p, displayed condition-dependent decreases. Notably, MBRT at 10 Gy elicited the broadest differential miRNA response, with a larger number of significantly dysregulated EV-associated miRNAs compared with the other irradiation conditions. At matched mean dose, direct CRT–MBRT differences remained limited, with miR-497-3p being the only miRNA reaching statistical significance in the direct modality comparison.

Although miR-497-3p was decreased relative to controls in irradiated groups, its reduction differed between modalities, resulting in significant differential expression in the direct CRT–MBRT comparison.

Together, these data indicate that (i) F98 sEVs cargo is unchanged at the applied doses, (ii) RG2 exhibits a limited set of modality-specific miRNA shifts, and (iii) non-cancerous astrocytes display a convergent irradiation response with minor CRT–MBRT divergence. Biological implications of individual miRNAs (e.g., *miR-374-5p*) and the limited annotation of certain features (e.g., *miR-6319*) are considered in the “Discussionsection.

Differential expression analysis was performed using *n* = 3 independent biological replicates per condition, and *p* values were corrected for multiple testing using the Benjamini–Hochberg false discovery rate (FDR) method.

Differential expression analysis was performed using *n* = 3 independent biological replicates per condition, and *p* values were corrected for multiple testing using the Benjamini–Hochberg false discovery rate (FDR) method.

A summary heatmap integrating significantly altered EV-associated miRNAs across irradiation conditions is shown in Supplementary Figure S1.

## Discussion

Given the strong preclinical confirmation that MBRT achieves effective tumor control while preserving normal tissue [37, 39–41, 43, 45, 46], this study provides, to our knowledge, the first molecular characterization of sEVs miRNA cargo following MBRT compared with conventional radiotherapy (CRT).

Results indicate that spatially modulated irradiation is associated with remodeling of EV miRNA cargo, suggesting altered EV signaling potential, consistent with the hypothesis that EVs may contribute to cohort- and bystander-like effects in spatially fractionated radiotherapy. Extracellular vesicles are increasingly recognized as key mediators of radiation-induced non-targeted effects because they can transfer bioactive molecules, such as miRNAs, proteins, and lipids, between irradiated and non-irradiated cells. Ionizing radiation alters EV biogenesis and cargo composition through DNA damage response pathways involving p53-dependent mediators such as TSAP6 and Rab GTPases, thereby influencing downstream signaling in recipient cells [57]. Notably, EV-associated miRNAs are selectively packaged rather than passively incorporated, highlighting an active regulatory mechanism [7]. EVs can thus play a role in inflammation, angiogenesis, immune responses, DNA repair, and tissue remodeling, all central components of radiation response through these mechanisms [58–60].

### Differential sEVs-miRNA Responses in Tumor and Normal Cells

Our results reveal marked differences in extracellular vesicle miRNA responses between tumor and normal cells following irradiation. Across the two glioma cell lines (F98 and RG2), no common EV-miRNA signature emerged at 24 h post-irradiation, and responses remained largely cell-line specific. Such differential responses may partly reflect intrinsic molecular differences affecting EV biogenesis and cargo loading, potentially involving p53-regulated pathways such as TSAP6- and Rab-dependent secretion mechanisms [57]. In F98 cells, no significant alterations in EV-associated miRNAs were detected under the conditions tested, whereas RG2 cells exhibited a limited set of modality- and dose-dependent changes, including modulation of miR-151-5p, miR-6319, miR-374-5p, and miR-466c-5p. Although some of these miRNAs remain poorly annotated, these findings suggest that early EV cargo remodeling in tumor cells is modest, heterogeneous, and likely influenced by intrinsic molecular characteristics, including differences in radioresistance and genetic background.

In contrast, astrocyte-derived sEVs displayed robust irradiation-associated miRNA remodeling, with partially overlapping signatures between CRT and MBRT at matched mean doses, although MBRT at 10 Gy elicited the broadest differential response. Several enriched miRNAs were consistent with stress adaptation, hypoxia-related signalling, metabolic regulation, and tissue-remodelling pathways, including repeated modulation of miR-210-3p across multiple irradiation conditions [61]. Other recurrently enriched miRNAs in our dataset, including miR-203b-3p and miR-672-3p, may also contribute to adaptive extracellular signalling, although their specific functional roles in irradiation-associated EV responses remain less well defined. More broadly, irradiation-associated EV-miRNA remodeling has been reported in other experimental and clinical settings [60, 62]. In contrast, miR-122b and miR-486 showed robust depletion throughout all irradiation settings, whereas decreases in miR-148a-5p were more condition-specific. Notably, MBRT at 10 Gy produced the broadest EV-miRNA response profile among all irradiation conditions tested.

Together, these observations indicate that EV-mediated responses to irradiation differ fundamentally between tumor and non-tumor cells: whereas glioma cells exhibit limited and heterogeneous early EV-miRNA modulation, astrocytes mount a pronounced and consistent vesicular response. This suggests that non-tumoral cells may contribute substantially to extracellular signalling dynamics following spatially modulated irradiation and could play an important role in shaping tissue-level adaptive responses after MBRT.

### Implications for Cohort Effects and Spatially Modulated Intercellular Communication

Non-targeted radiation effects, including bystander and cohort effects, arise when irradiated cells influence neighboring or distant cells through secreted factors such as EVs. The spatial heterogeneity of MBRT introduces peak and valley dose regions, potentially generating gradients of EV-mediated signaling. EVs released from cells receiving different radiation doses may carry distinct molecular signals that coordinate tissue-level responses across the irradiated volume [63].

Our observation that MBRT primarily altered EV cargo rather than EV concentration at early time points suggests that selective miRNA loading, rather than bulk EV production, is a critical regulatory mechanism. This aligns with previous reports showing that radiation-induced functional effects are primarily mediated through cargo modulation rather than vesicle quantity [22–24].

Similarly, prior studies have demonstrated that radiation modality can influence EV biology, with proton irradiation reported to alter EV production and downstream signalling compared with conventional photon irradiation [62, 64].

Together, these findings support a model in which spatial dose distribution shapes intercellular communication networks via EV cargo modulation. In normal tissue, this may promote vascular stability, immune regulation, and tissue repair. Notably, some irradiation-enriched EV-associated miRNAs identified here, such as miR-25-3p, have previously been implicated in regulating vascular permeability, endothelial remodelling, and angiogenic signalling, supporting a potential role for EV cargo in tissue-level adaptive responses after irradiation [65].

Beyond their role in maintaining neural homeostasis, astrocytes may also influence long-term tumor evolution after irradiation through EV-mediated communication. Astrocyte-derived extracellular vesicles have been shown to regulate glioma cell behavior by transferring miRNAs, metabolites, and signaling molecules capable of modulating proliferation, migration, stemness, and treatment resistance. In the post-irradiation setting, reactive astrocytes could therefore contribute to shaping a microenvironment that is either tissue-protective or, conversely, supportive of residual tumor cell survival and recurrence. This possibility is particularly relevant in glioblastoma, where tumor relapse is strongly influenced by microenvironmental interactions within the peritumoral niche. In this context, the pronounced remodeling of astrocyte-derived EV miRNA cargo observed here may not only reflect adaptive tissue signaling but could also represent a mechanism through which irradiated normal brain cells indirectly affect tumor repopulation dynamics. Future studies should therefore investigate whether irradiation-induced astrocyte EVs promote anti-tumor immune surveillance and tissue repair, or alternatively facilitate recurrence-promoting niche signaling depending on the biological context.

However, astrocyte-derived EV signaling is likely context-dependent: while it may contribute to tissue protection and restoration of homeostasis, astrocyte-derived vesicles can also, under certain microenvironmental conditions, support glioma cell survival, invasion, and recurrence through pro-tumorigenic niche signaling [16, 29, 59].

Future studies should therefore investigate whether irradiation-induced astrocyte EVs promote anti-tumor immune surveillance and tissue repair, or alternatively facilitate recurrence-promoting niche signaling depending on the biological context. Functional studies will therefore be required to determine whether irradiation-induced astrocyte EV remodeling favors protective tissue adaptation, tumor-supportive signaling, or a context-dependent balance between both outcomes.

### Translational Implications: EV-Associated miRNAs as Candidate Biomarkers and Mechanistic Mediators

From a translational perspective, EV-associated miRNAs may ultimately prove informative as minimally invasive indicators of radiation response, given that EVs circulate in biological fluids such as blood and cerebrospinal fluid and that their molecular cargo can reflect cellular physiological states [8, 28]. In this context, the identification of irradiation-associated EV-miRNA signatures raises the possibility that EV profiling could, in the future, contribute to monitoring biological responses to treatment and refining our understanding of modality-dependent radiation effects [60].

However, the present findings should be interpreted cautiously. The current study was performed in simplified in vitro rat monoculture systems, at a single early time point, and without functional validation of EV uptake or downstream biological activity in recipient cells. Therefore, although the observed EV-miRNA remodeling suggests altered signaling potential following irradiation, direct mechanistic consequences and translational applicability remain to be established.

Rather than providing definitive biomarker candidates or demonstrating causal mediators of the therapeutic advantages of MBRT, our results identify EV-miRNA remodeling as a previously unrecognized molecular response to spatially modulated irradiation that generates hypotheses for future studies. Validation in human-derived models, longitudinal experimental designs, and functional EV transfer assays will be necessary to determine whether EV-associated miRNAs can serve as robust biomarkers or biologically relevant mediators of responses to spatially fractionated radiotherapy.

## Limitations and future directions

Several limitations should be considered when interpreting the present findings. First, this study was conducted in simplified in vitro monoculture systems using rat-derived cell lines, which do not fully recapitulate the cellular complexity, heterogeneity, and microenvironmental interactions present in glioblastoma in vivo. Consequently, the translational relevance of the observed EV-miRNA signatures to human disease remains to be established.

Second, analyses were restricted to a single early post-irradiation time point (24 h), whereas extracellular vesicle release and cargo composition are dynamic processes that likely evolve over time. Longitudinal profiling will therefore be necessary to capture the temporal kinetics of irradiation-induced EV remodeling.

Third, although differential EV-miRNA cargo remodeling was identified, no functional assays were performed to directly assess EV uptake, transfer efficiency, or downstream biological effects in recipient cells. As a result, changes in EV cargo reported here should be interpreted as indicative of altered signaling potential rather than demonstrated functional modulation of intercellular communication. Future studies using multicellular co-culture systems, recipient-cell assays, and in vivo models will be required to establish mechanistic relevance.

Fourth, biological interpretation of dysregulated miRNAs remains inference-based, relying on previously reported functional associations in the literature rather than direct pathway-specific experimental validation in the present model system. Therefore, associations with DNA damage response, hypoxia-related adaptation, immune regulation, or tissue-protective signaling should be viewed as hypothesis-generating rather than definitive mechanistic evidence.

Finally, extracellular vesicle studies are sensitive to pre-analytical variables, including culture conditions during conditioned-medium collection, which may influence vesicle release and cargo composition. Although all experimental and sham-irradiated control groups were processed identically in this study, careful standardization of these variables remains essential for future mechanistic and translational investigations.

Future work should therefore incorporate human-derived models, longitudinal experimental designs, functional EV transfer assays, and complementary multi-omics approaches to better define the biological and translational significance of EV remodeling following spatially modulated irradiation.

## Conclusion

This study provides an initial exploratory characterization of EV-miRNA remodeling following CRT and MBRT in rat glioma and astrocyte models. Whereas glioma cells showed limited and heterogeneous responses, astrocytes displayed robust EV cargo remodeling with signatures consistent with cellular stress adaptation. These findings generate hypotheses regarding the role of EV-associated miRNAs in spatially modulated radiation responses, warranting future functional and translational investigation.

## Supplementary Information

Below is the link to the electronic supplementary material.

ESM 1 (DOCX 1.28 MB)

## Data Availability

The data will be available upon request to the corresponding author.
