# Supplementary material for: Spatially Modulated Irradiation Alters Extracellular Vesicle MicroRNA Cargo in Rat Glioma and Astrocyte Models
Source: Mol Neurobiol. 2026 Jun 11;63(1):689. doi: 10.1007/s12035-026-05988-5 (PMC13260173; doi:10.1007/s12035-026-05988-5)
Supplement: Supplementary file 1 — (DOCX 1.28 MB) [file 12035_2026_5988_MOESM1_ESM.docx]

**Supplemental materials**


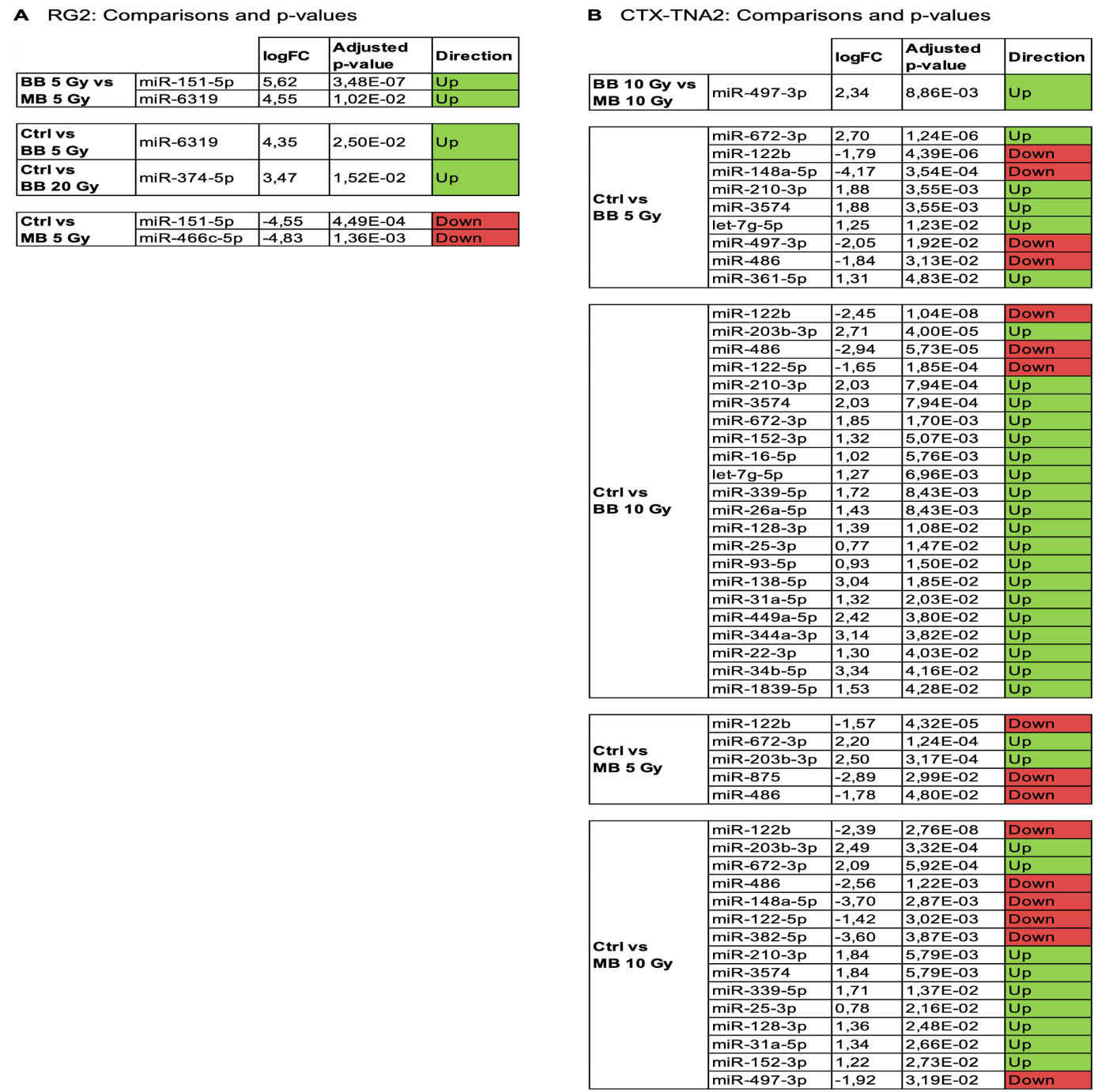


**Table S1**: Table summarizes miRNAs significantly altered across irradiated conditions, in (**A**) RG2 cell line, and in (**B**) CTX-TNA2 cell line. Values shown are logFC and adjusted p-values, as well as the direction of the dysregulation.


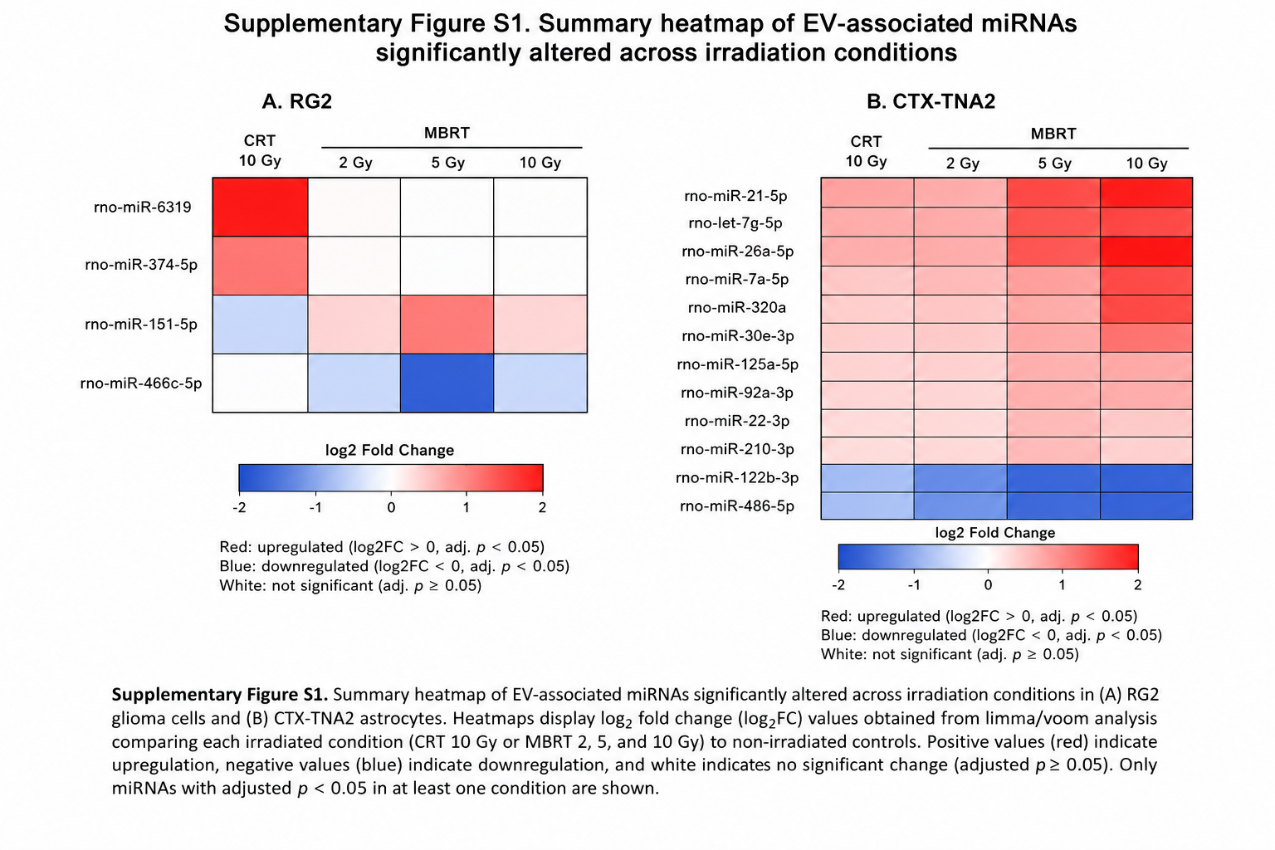


**Supplementary Figure S1. Summary heatmap of significantly dysregulated EV-associated miRNAs across irradiation conditions.** Heatmaps summarize the log2 fold-change (log2FC) of EV-associated miRNAs significantly altered following irradiation in (A) RG2 glioma cells and (B) CTX-TNA2 astrocytes, relative to non-irradiated controls. Comparisons include CRT (10 Gy) and MBRT (2, 5, and 10 Gy) conditions. Positive values (red) indicate upregulation, negative values (blue) indicate downregulation, and white indicates no statistically significant change (adjusted p ≥ 0.05). Only miRNAs reaching statistical significance (adjusted p < 0.05) in at least one condition are shown. Differential expression was determined using the limma/voom framework on n = 3 independent biological replicates per condition.
